# MMNet: A Mixing Module Network for Polyp Segmentation

**DOI:** 10.3390/s23167258

**Published:** 2023-08-18

**Authors:** Raman Ghimire, Sang-Woong Lee

**Affiliations:** 1 Pattern Recognition and Machine Learning Lab, Department of IT Convergence Engineering, Gachon University, Seongnam 13557, Republic of Korea; ghimirermn@gmail.com; 2 Pattern Recognition and Machine Learning Lab, Department of AI Software, Gachon University, Seongnam 13557, Republic of Korea

**Keywords:** polyp segmentation, transformer, computational complexity, depth-wise and 1 × 1 convolution, mixing module

## Abstract

Traditional encoder–decoder networks like U-Net have been extensively used for polyp segmentation. However, such networks have demonstrated limitations in explicitly modeling long-range dependencies. In such networks, local patterns are emphasized over the global context, as each convolutional kernel focuses on only a local subset of pixels in the entire image. Several recent transformer-based networks have been shown to overcome such limitations. Such networks encode long-range dependencies using self-attention methods and thus learn highly expressive representations. However, due to the computational complexity of modeling the whole image, self-attention is expensive to compute, as there is a quadratic increment in cost with the increase in pixels in the image. Thus, patch embedding has been utilized, which groups small regions of the image into single input features. Nevertheless, these transformers still lack inductive bias, even with the image as a 1D sequence of visual tokens. This results in the inability to generalize to local contexts due to limited low-level features. We introduce a hybrid transformer combined with a convolutional mixing network to overcome computational and long-range dependency issues. A pretrained transformer network is introduced as a feature-extracting encoder, and a mixing module network (MMNet) is introduced to capture the long-range dependencies with a reduced computational cost. Precisely, in the mixing module network, we use depth-wise and 1 × 1 convolution to model long-range dependencies to establish spatial and cross-channel correlation, respectively. The proposed approach is evaluated qualitatively and quantitatively on five challenging polyp datasets across six metrics. Our MMNet outperforms the previous best polyp segmentation methods.

## 1. Introduction

Among cancer-related deaths [1], colorectal cancer is one of the leading causes. Colorectal cancer often begins with a polyp which can be benign, non-cancerous, or malignant. If not treated, some of these polyps can potentially turn into life-threatening cancer [2]. Thus, early detection and identification of such polyps is of utmost importance. Currently, colonoscopy is one of the most prevalent methods to screen cancerous polyps, and such screening has to be carried out at regular intervals to ensure no colon cell growth [3]. Since colonoscopy treatment involves humans, there is always a chance of human error, so computer-aided techniques are used to reduce such errors. However, even with a computer-aided process, the segmentation and detection of colons are difficult tasks. Polyps vary by their size, shape, and color intensity. The polyp cells and their separating boundary are generally blurred and lack distinction to separate them from their surrounding mucosa, making them difficult to segment.

Early polyp segmentation methods involved hand-crafted feature methods [4,5]; however, such methods showed very low accuracy and failed to capture polyp heterogeneity. To help correctly detect polyps, computer-aided techniques such as fully convolutional networks (FCN) [6] were introduced. FCNs demonstrated comparative improvements in polyp segmentation [7] and introduced an encoder–decoder-based architecture, which turned out to be a decisive point in semantic segmentation. Motivated by this, U-Net [8] introduced skip connections into the symmetric encoder–decoder network. The encoder extracts features through a feature map with a sequence of down-sampling operations. The decoder then, with the use of skip connections, progressively concatenates the feature context from the encoder. Skip connections helped in retaining features as well as combining deep semantic and spatial information. The advent of U-Net led to the development of various types of networks [9,10,11]; however, one common characteristic among all of them was the inability to model long-range dependencies. Even with the usage of attention blocks or the varied use of skip connections, the inability of the network to build long-range dependencies was still present.

The introduction of transformers [12] into the visual domain thus played a significant role in building long-range dependencies. With the advent of ViT [13], transformer models could finally be used in the visual domain for global context, similar to NLP. The ViT model obtained competitive results on ImageNet when pre-trained on a large corpus of image data. Although transformer models could leverage the global context, they needed a large amount of data to generalize. Furthermore, they were computationally expensive and lacked the inherent inductive bias present in CNN models. In order to address these challenges, transformers coupled with CNNs were brought into practice. Networks like TransUNet [14], Polyp-PVT [15] and Medical Transformer [16] leveraged both the transformer’s and CNN’s capabilities, providing an improved performance. However, even when coupled with convolutional networks, the computational cost with repeated application of self-attention mechanisms was still inherent in hybrid transformer networks. To reduce the computational cost while maintaining the output performance, mixing networks [17,18] were introduced. They repeatedly apply either MLP layers or convolution layers to model long-range dependencies. Specifically, the network is divided into two subtasks of mixing features across spatial and feature channels. Contrary to how transformer networks used self-attention mechanisms with a higher computational cost, mixer networks achieved a similar performance with a lower computational cost. Furthermore, unlike transformers’ complexity and arduous network design, mixing networks were easier to understand and interpret.

This paper proposes a novel multi-stage polyp segmentation network called Mixing Module Network (MMNet). Moreover, this paper introduces the integration of a feature mixing approach for generalized polyp segmentation. The idea behind mixing networks is similar to how attention networks are used but with a lower computational cost. Mixing networks are introduced into the network to give a per-pixel weight to the polyp image. We apply a series of convolutional operations similar to mixing network mixed features by the repeated application of multi-layer perceptrons or convolution layers. Series of repeated convolutional operations are chosen over MLPs primarily because MLP mixers [17] do not mix features across spatial locations like convolutional mixers [18,19] do. In our method, a mixing module replaces the final stage of the conventional U-net architecture. Here, a CNN model pre-trained on PVTv2 [20] is used as a feature extractor, and the features of the encoder are aggregated to form a global feature map. The mixing module then takes this resultant global feature map as the input, generating a recalibrated feature map. The recalibrated feature map from the mixing module is then applied to the global feature map to generate a segmented mask. The contributions of this paper are as listed below:

- We propose a multi-stage transformer coupled mixing network to achieve improved performance in polyp segmentation. This method aims to improve long-range dependencies with a reduced computational cost.
- We introduce a feature mixing module with which the global feature map generated on the encoding region is further enhanced by highlighting the necessary information and suppressing the unnecessary information.
- We validate our MMNet extensively with five different datasets. Our network can accurately segment polyps and thus consistently outperforms the previous best methods.

The remaining sections of this paper are arranged as follows. Related work is discussed in Section 2, followed by the proposed method in Section 3. The experiments and results are in Section 4, Section 5 includes the discussion and future work, and finally, Section 6 contains the concluding remarks.

## 2. Related Works

In this section, we briefly explain the related works in four parts. In the first and second parts, we review deep learning methods for image segmentation tasks followed by the attention mechanisms used in them. In the third part, we review feature selection approaches used in polyp segmentation, and finally, in the fourth part we review transformer and mixer networks.

### 2.1. Deep Learning for Image Segmentation

Long et al. [6] proposed a fully convolutional network which was pioneering in segmentation architecture. The authors utilized existing classification models by switching the final feed-forward layer with 1 × 1 convolutions. To match the result with the input size, the final result would then be up-sampled with skip connections, which would concatenate the features from lower layers. After FCN, U-Net [8] was proposed for image segmentation. U-Net uses a convolving and expanding architecture, simply put, an encoder–decoder architecture, to segment images. To recapitulate the lost information, the decoder uses skip connections, and unlike an FCN, where feature maps are summed, U-Net models concatenate them. U-net models are the preferred models for most image segmentation tasks. UNet++ [9] introduced skip connections with redesigned pathways. The redesigned skip pathways aimed at combining semantic information at varying scales. However, it is difficult to model long-range dependencies with UNet models, even with redesigned skip connections.

### 2.2. Attention Mechanism in Image Segmentation

To further improve the generalization capability of U-Net, Attention U-Net [21] introduced attention gates. Attention gates are used to suppress feature activations in irrelevant regions and highlight necessary salient features. This then improved the model’s sensitivity and, thus, dense label prediction accuracy. Such attention gates could be fit into standard CNN architectures with minimal computational overhead costs. ResUNet++ [11] introduced SENet [22] in the encoder–decoder section to model the interdependencies between the channels. Furthermore, attention blocks were also utilized to enhance the quality of features. SENet was used in the U-Net network’s encoder section, whereas attention blocks were used in the decoder section. DANet [23] appended two types of attention modules, one in the spatial and the other in the channel dimension. A position attention module aggregated the features at each position to learn cross-sectional interdependencies, whereas the channel attention module integrated the associated features among all channel maps by emphasizing the interdependent channel maps. Additionally, the outputs of two separate attention modules were be combined to enhance feature representation further. EMANet [24] introduced an attention mechanism as an expectation maximization tool and used an iterative mechanism to compute attention maps. Such attention maps were computed based on the expectation-maximization algorithm. Furthermore, such representation was robust and computationally efficient.

### 2.3. Feature Selection Approach in Polyp Segmentation

Using boundary cues, recent works such as SFA [25], PraNet [26], MSNet [27], and UACANet [28] have focused on retrieving the separation boundary between a polyp and its adjacent boundary. Selective feature aggregation (SFA) [25] utilizes boundary and area constraints using a standard encoder and dual decoders to aggregate the important features selectively. In this paper, feature aggregation takes place with the help of embedding selective kernels placed in the convolutional layers and by utilizing three concatenations between the encoder and decoder layers. This paper also introduces a new boundary-sensitive loss function to determine the dependency between the area and the boundary branch. A parallel reverse attention network (PraNet) [26] utilizes a reverse attention module to generate the boundary cues. Prior to this, PraNet also generates a guidance area by aggregating features from the high-level layers. A reverse attention module is then applied to these aggregated features to calibrate the misaligned predictions.

SFA and PraNet generate redundant information between the adjacent layers, resulting in an inaccurate polyp segmentation. Thus, to reduce such redundancy, networks like MSNet [27] and UACANet [28] have introduced various additional methodologies. MSNEt introduced a multi-scale subtraction network where difference features between adjacent encoders are produced with the help of subtraction units. Such subtraction units are placed pyramidically at different levels to obtain rich multi-scale difference features. UACANet furthermore utilized the existing PraNet architecture by strengthening the feature encoding region and introducing an uncertainty context attention module. This module helped to accurately segment polyps by focusing more on saliency maps.

### 2.4. Transformer and Mixing Models

In the natural language processing domain, a transformer [12] showed a remarkable improvement over prevalent state-of-the-art (SOTA) architectures. Motivated by this, a transformer suited for the vision domain, ViT [13], was introduced. Since the data structures used in NLP and vision are entirely different, a novel architecture had to be designed to integrate the transformer architecture. Furthermore, using image patches as flattened vectors, ViT obtains exemplary results on image recognition, provided it was pre-trained on massive datasets such as ImageNet-22k. However, this also means that ViT requires a large corpus of data to pre-train on. DeiT [29] showed that the transformer architectures could be used on medium-sized datasets with a distillation approach, where a CNN acts as a teacher model to train the transformer model. In this way, DeiT injects the inductive bias into the model that ViT was missing. This led to a spark of change in the visual domain. In the medical domain, TransUNet [14] was the first work to introduce a transformer model for medical image segmentation on a synapse multi-organ segmentation dataset. Precisely, TransUNet introduced a transformer as an embedding block for global self-attention. The authors used a combined CNN–transformer network to capture the global context and spatial features from the transformer and convolutional blocks. Medical Transformer [16] is also based on the classical U-Net architecture, where a transformer is placed after the encoding section to obtain the global context of features. The decoder section then sequentially upsamples the input from the transformer and encoder through skip connections to obtain the segmented image. The basic idea behind the transformers in vision is to model the long-range dependencies applying repeated self-attention blocks. The only downside of including a transformer is the computational complexity with the repetitive application of self-attention blocks.

Mixing networks like MLP-mixer [17] and Convmixer [18] were introduced to alleviate such complexity issues. MLP-mixer consists of two different layers: the first layer mixes features across image patches, and the second layer mixes features spatially. Similarly, in the case of Convmixer, repeated convolution operations are carried out to establish spatial and cross-channel correlation. The repeated application of MLPs or convolutions works similarly to the repeated application of the self-attention mechanism in transformer networks but with a reduced computational cost (Algorithm 1).
**Algorithm 1** Pseudo-Code for Mixing Module Network**Input:** Polyp Image **I**, Ground Truth **G****Output:** Predicted Mask, **M**  1:**for** epoch n = 1, 2 … **do**  2:  Feature extraction using Pyramid Vision Transformer as Encoder, E  
$O_{Encoder}=E_{Input}\left(I\right)=F_{1},F_{2},F_{3},F_{4}$  3:  Application of FEB Module on Output from Encoder  
FEB{1,2,3,4}=FEB(O(Encoder))  4:  Global Feature Map generation using PPD and only on FEB{2,3,4}  
$G\_Feature=PPD\left\{R_{2},R_{3},R_{4}\right\}$  5:  Patch the global feature map and pass it to the mixing module network (Equations (2) and (3))  6:  Compute scores by applying sigmoid on mixing module output, σ(MMOutput)  7:  Compute output mask by applying σ(MMOutput) on global feature map  
M=σ(MMOutput)xG_Feature  8:  Optimize the network using loss function  Ł = *LIOU* + *LBCE*  9:**end for**

## 3. Methodology

The details of the network architecture are provided in this section. The first part consists of an overview of the model. The second part consists of a description of feature aggregating components. The feature mixing module is explained in the third part, and finally, in the fourth part, the loss function is explained.

### 3.1. Overview of the Model

**Figure 1:**
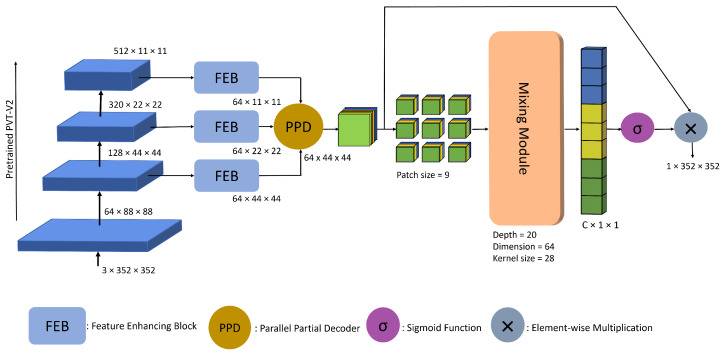
The architecture of our proposed approach, MMNet, which consists of FEB, PPD and a mixing module for polyp segmentation.

The network design can be seen in Figure 1. The figure shows that the encoding region uses a pre-trained Pyramid Vision Transformer (PVTv2) [20] as a feature extractor. Polyp images are passed to the transformer, which produces features at different scales. Such features are then passed through the feature-enhancing blocks, FEB, to enhance feature representation and robustness. A modified parallel partial decoder, PPD [30], is used to produce a global feature map using three higher-level features. The global feature map is divided into patches before being fed to the mixing module. The mixing module is used to mix features both spatially and across channels. This sort of feature mixing helps with the interaction of features at different scales and, thus, ultimately helps highlight informative features and suppress the less useful ones. The output of the mixing module is then passed through a sigmoid function and then applied to the global feature map to produce a subsequent mask. A detailed explanation is listed below.

### 3.2. Feature Enhancer and Parallel Partial Decoder

**Figure 2:**
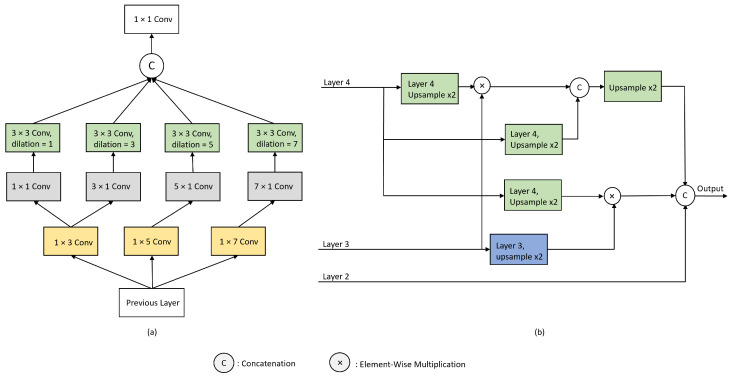
(**a**) shows the feature-enhancing block (FEB) and (**b**) shows the modified parallel partial decoder (PPD).

Conventional U-Net architectures carry out feature aggregation in all the encoder layers. However, according to Wu et al. [30], the lower layers of the encoder are computationally expensive and have a significantly lower contribution to the model performance. In contrast, the higher layers of the encoder contain refined feature representation. Following this, we aggregate only the top layer features of an encoder to obtain the global feature map. Before obtaining the global feature map, the encoder output is also passed through feature-enhancing blocks (FEBs) to further strengthen the feature representations. These FEBs are modified versions of RFB blocks [31] with added dilation rates to increase the spatial resolution. The FEBs use progressively larger filter sizes to obtain refined feature representations, as shown in Figure 2a. Particularly, for an input image of size *H* × *W*, four levels of features, $S_{m}$, *m* = {1, 2, 3, 4} from the pretrained Pyramid Vision Transformer (PVTv2) [20] backbone are extracted. Furthermore, based on PPD, as seen in Figure 2b, the lower-level features, i.e., $S_{m}$, *m* = {1}, are discarded and only the higher-level feature, i.e., $S_{m}$, *m* = {2, 3, 4}, are utilized. These three feature maps are strengthened separately following the FEB module and then aggregated using the modified parallel partial decoder, PPd, to obtain a global feature map.

### 3.3. Feature Mixing Module

**Figure 3:**
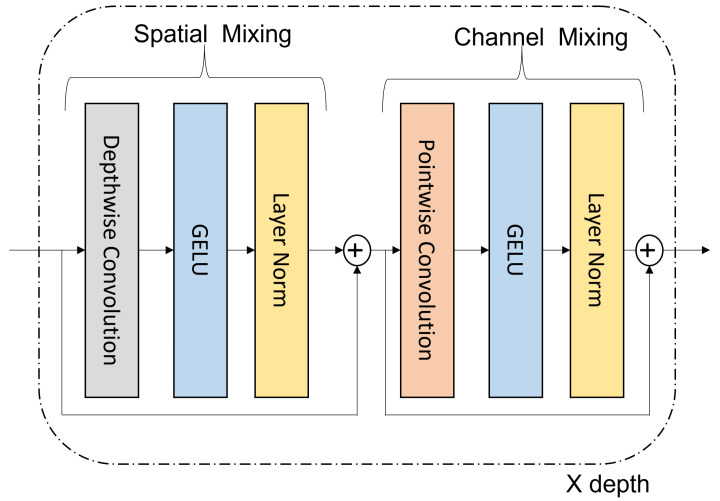
Mixing module for recuperating long-range dependencies.

In prevalent convolutional networks, different attention mechanisms have been used to leverage distant features in vision and NLP applications. Attention mechanisms have achieved significant improvements in modeling long-range dependencies. Inspired by this, and especially the squeeze and excitation network [22], where the network assigns per-pixel weights by squeezing features across channels, and the recent advent of the Convmixer architecture [18], we propose a feature mixing module to establish pixel-level long-range dependencies. Given a feature map × $\in R^{H\times W\times C}$ with *H* × *W* as the spatial resolution and *C* as the number of channels, the role of the feature mixing module is first to establish a correlation between spatial and channel dimensions and secondly to calculate the per channel weights to be applied to the feature map. Using a large kernel size helps to mix features across spatial and channel regions, and average pooling establishes the per channel weights. As seen in Figure 3, we use a depth-wise and 1 × 1 convolution to establish spatial and cross-channel correlation, respectively.

We first perform patch embeddings followed by repeated application of convolution layers. For a feature map with $C_{in}$ channels, the patch embeddings with patch size *p* and embedding dimension h can be expressed as:

(1) $$\begin{array}{c} z_{o}=\mathrm{LN}\left(\sigma \left\{\mathrm{Conv}2D\left(\dim,\dim,\mathrm{kernel}\;\mathrm{size}=p,\mathrm{stride}=p\right)\right\}\right) \end{array}$$

As mentioned above, the mixing module utilizes the Convmixer architecture [18]; however, with specific explicit changes. In the Convmixer architecture, the authors utilized batch normalization [32] throughout the network and mentioned a relatively small percentage improvement over layer normalization (LN) [33]. In our case, where the batch size is relatively small (16) and the image size is large (352 × 352), using batch normalization would be less practical than layer normalization. Hence, unless otherwise stated, we used layer normalization throughout the mixing module. Furthermore, we added one more residual connection compared with the Convmixer architecture to propagate features when establishing spatial and cross-channel correlation. Mathematically, the mixing module with depth-wise and 1 × 1 convolution can be expressed as follows:

(2) $$z_{l}=\mathrm{LN}\left(\mathrm{GELU}\left\{\mathrm{DepthwiseConv}2D\left(z_{l-1}\right)\right\}+z_{l-1}\right)$$

(3) $$z_{l+1}=\mathrm{LN}\left(\mathrm{GELU}\left\{\left(1\times 1\;\mathrm{Conv}\right)\left(z_{l}\right)\right\}+z_{l}\right)$$

where LN [33] is layer normalization and GELU [34] is an activation function. We repeat the same operation x times to obtain a final feature map. The feature map is then averaged and pooled to obtain per channel weights. The per-channel weight is then passed through a sigmoid function before being applied to the global feature map to obtain the final segmentation mask.

### 3.4. Loss Function

We use a mixture of binary cross entropy (BCE) loss and intersection over union (IOU) loss as a loss function. In the segmentation task, BCE is used to compute pixel-level classification, whereas IOU is used to measure the similarity between the predicted and ground truth regions in the image. They are both given in Equations (4) and (5) as follows:

(4) $$\begin{array}{c} L_{BCE}=-\sum_{e,p}\left[R\left(e,p\right)\log\left(\hat{R}\left(e,p\right)\right)+\left(1-R\left(e,p\right)\right)\log\left(1-\hat{R}\left(e,p\right)\right)\right] \end{array}$$

(5) $$L_{IOU}=1-\frac{\sum_{e,p}R\left(e,p\right)\hat{R}\left(e,p\right)}{\sum_{e,p}\left[R\left(e,p\right)+\hat{R}\left(e,p\right)-R\left(e,p\right)\hat{R}\left(e,p\right)\right]}$$

where *R*(*e*,*p*)∈{0, 1} is the ground truth label of the pixel (e,p) and $\hat{R}\left(e,p\right)$ is the predicted probability of the output. In segmentation tasks, BCE is a widely used loss function; however, as mentioned in [35], it has three major drawbacks. Firstly, it discards the global structure and only calculates the loss for pixels independently. Secondly, foreground pixel loss is diluted in images with a dominant background. Finally, BCE takes all pixels with an equal importance. Motivated by this, we use the hybrid loss function, which focuses more on hard pixels. Adding IOU loss helps to optimize the global structure instead of focusing on a single pixel. The final loss function $L_{total}$ is computed as follows:

(6) $$\begin{array}{c} L_{total}=L_{BCE}+L_{IOU} \end{array}$$

## 4. Experiments and Results

### 4.1. Datasets

We tested our model across five datasets: Kvasir [36], CVC-ClinicDB [37], CVC- ColonDB [38], CVC-300 [39] and ETIS [40]. The Kvasir dataset consists of 1000 polyp images and the corresponding ground truth. The resolution of the images in this dataset varies from 332 × 487 to 1920 × 1072. CVC-ClinincDB consists of 612 images, and the size of the images is 384 × 288. CVC-300 is a test dataset from Endoscene. Following PraNet [26], we used 60 of its images as a test dataset. CVC-ColonDB consists of 380 images, and the size of the images is 574 × 500. The ETIS dataset consists of 196 images with an image size of 1225 × 966. The size of polyps in this dataset is primarily small, which makes generalization using this dataset difficult.

Among the five datasets, Kvasir and CVC-ClinicDB were used for training and testing, whereas the remaining were strictly used for evaluating generalization on unseen data. To maintain a lack of bias when comparing the results, we followed the training and testing dataset partition used in PraNet [26].

### 4.2. Evaluation Metrics

For quantitative evaluation, we adopted several widely popular evaluation metrics such as mDice, mIOU, $F_{\beta }^{\omega }$ [41], $E\phi^{\max}$ [42], $S_{\alpha }$ [43] and MAE. The weighted F-measure ($F_{\beta }^{\omega }$) offers a unified solution for evaluating binary and non-binary maps based on either pixels or structure. The E-measure or enhanced-alignment measure ($E\phi^{\max}$) is used to account for both pixel and image level properties. S-measure (Sα) is used to measure the similarity between predictions and ground truths, and finally the MAE metric is used to evaluate differences based on pixel-level values. In all the listed metrics, except MAE, a higher value indicates good results and a lower value indicates a poor performance.

### 4.3. Implementation Details

**Figure 4:**
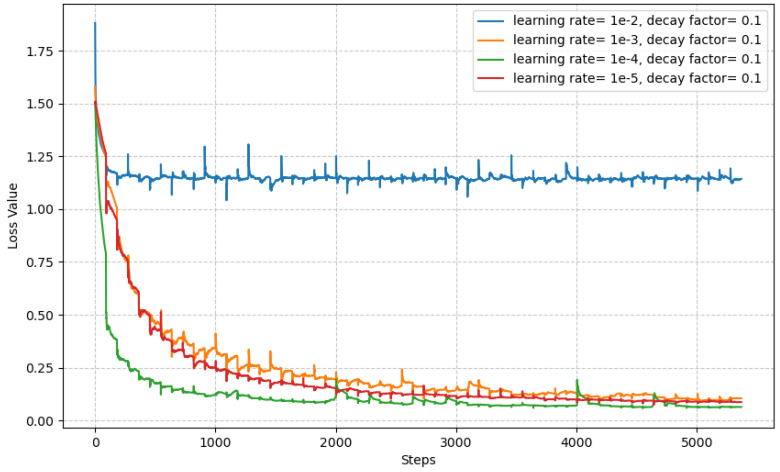
Loss plot for different learning rates.

We implemented our MMNet in the Pytorch framework (https://pytorch.org/). We used PVTv2 [20] as an encoder backbone. To generate a global feature map, we used feature enhancing blocks, FEB, which have modified strides and added dilation rates in Receptive Field Blocks [31]. This is followed by a parallel partial decoder, PPD, which also has modified strides and added dilation rates to enlarge the spatial dimensions of the feature map. The mixing module is set with a depth of 20, dimensions of 64, a kernel size of 28 and a patch size of 9. We trained the model on a single Tesla V100 GPU (NVIDIA, Santa Clara, CA, USA). The images were rescaled to 352 × 352 during training and inference periods. We employed a multiscale training strategy {0.75, 1, 1.25}, accompanied by data augmentation techniques, which include probabilistic rotation up to 90 degrees, rescaling, as well as horizontal and vertical flips. The Adam optimizer [44] was used with the learning rate set to 1 ×$10^{-4}$ and a polynomial learning rate decay with a factor of 0.1. The network was trained for 60 epochs, and batch size was set to 16 unless otherwise specified. More details about the choice of learning rate can be seen in Figure 4.

### 4.4. Evaluation Results

The proposed network was evaluated on the five datasets mentioned above to illustrate its effectiveness. The quantitative result was compared against the best models, including U-Net++, SFA, PraNet, EU-Net, MSNet, UACANet-S, UACANEt-L and Polyp-PVT. To maintain a lack of bias, the results used for comparison are either calculated with their released code or provided by their respective authors. The quantitative results can be seen in Table 1 and Table 2. As mentioned previously, among the five datasets, only Kvasir and CVC-ColonDB datasets were used in the training scheme. Thus, most of the models perform well on the testing dataset. On the Kvasir-Seg dataset, our network has a dice score of 0.917 and an mIOU of 0.866. The dice score of our model is comparable to that of Polyp-PVT, the current best method, while the mIOU metric shows a slight improvement. Similarly, on the CVC-ClinicDB dataset, our network has a dice score of 0.937 and an mIOU of 0.889 which are similar to those of the current best method.

The challenging datasets are the unseen CVC-ColonDB, CVC-300 and ETIS. Via these datasets, we can determine the generalization capability of the applied approach. On the CVC-Colondb dataset, our network has a dice score of 0.812, which is a 0.4% improvement over the current best method, Polyp-PVT, and a 5% improvement over UACANet-L, the second best method. On the mIOU metric, our network exhibits a 0.001% improvement over Polyp-PVT and a 5% improvement over UACANet-L. On the CVC-300 dataset, UACANet-L has a 1% better metric than both Polyp-PVT and our network. Among these three datasets, ETIS is considered the most difficult to generalize due to the significant number of small-sized polyps. On this dataset, our network has a dice score of 0.807, which is a 2% improvement over Polyp-PVT and a 4.1% improvement over UACANet-L. On the mIOU metric, our network demonstrates 4.6% and 6.3% improvements over Polyp-PVT and UACANEt-L, respectively. On other metrics such as $F_{\beta }^{\omega }$, $S_{\alpha }$ and $E\phi^{\max}$, our applied approach shows significant improvements. Furthermore, when comparing the standard deviations of the mean dice scores obtained after training with different networks, as presented in Table 3, it is evident that there is minimal variation between our model and the compared models. This consistency in the results indicates that both models exhibit stability and reliability in their performance during evaluation.

**Figure 5:**
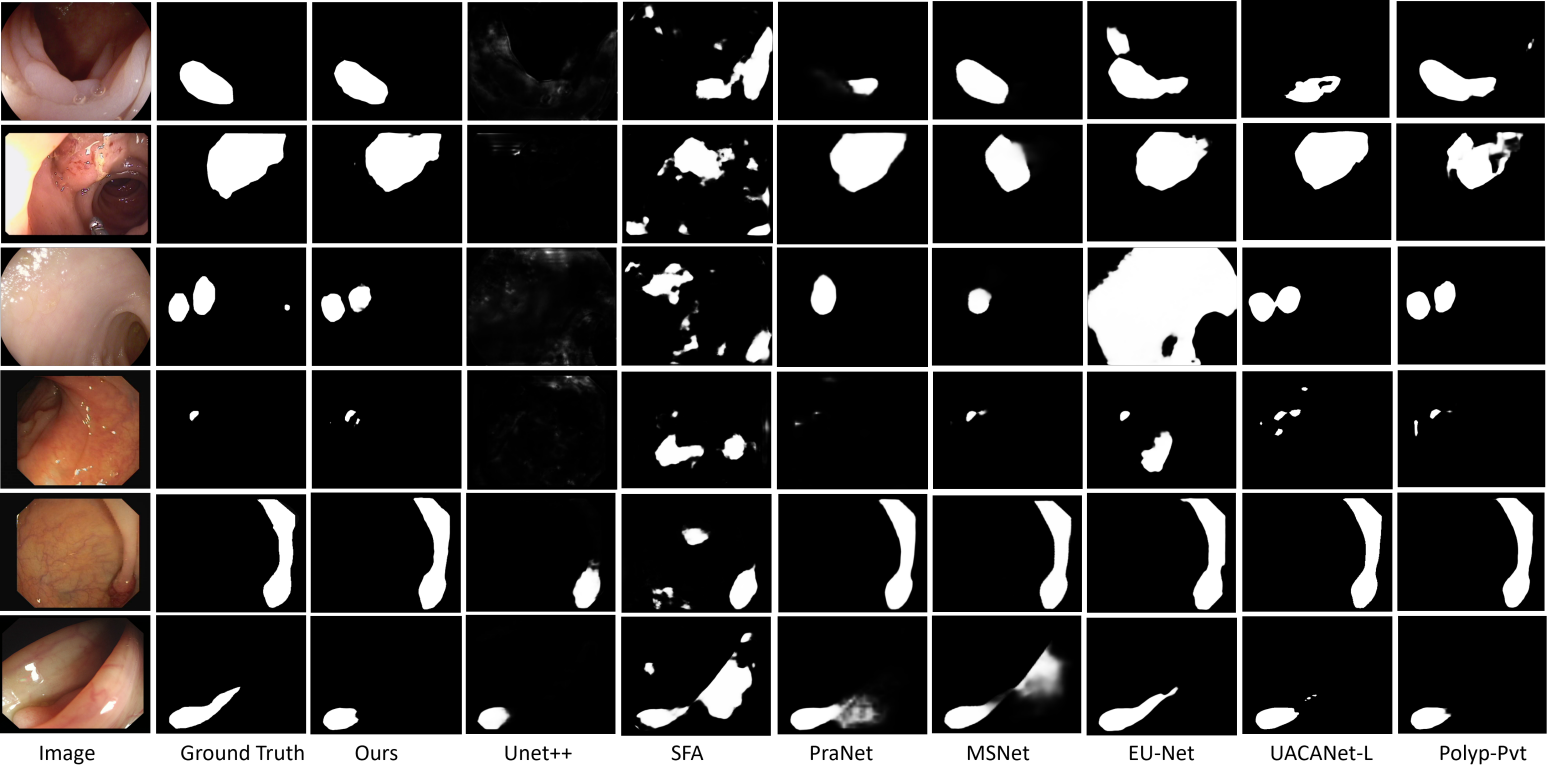
Qualitative comparison of outputs of different networks.

The output performance of the proposed model was further verified qualitatively in Figure 5. The performance was measured against several state-of-the-art (SOTA) architectures. In these comparisons, we evaluated the model’s performance on various polyp images with differing sizes and under varying light intensities. The results of the evaluation show that our network outperforms the previous best methods, consistently approaching ground truth accuracy across different types of polyp images. Whether dealing with large-sized, small-sized, or multi-polyp images, our model consistently achieves better results compared to existing approaches. Moreover, our network demonstrates impressive polyp segmentation capabilities in images exhibiting challenging conditions, such as varying contrast, reflections or the presence of tiny objects. These challenging scenarios have historically posed difficulties for traditional segmentation methods. However, our model’s performance surpasses those of previous methods, showcasing its robustness and adaptability in handling complex and diverse polyp images. The collective findings from this extensive evaluation further confirm the validation and efficacy of our proposed network in the polyp segmentation task.

### 4.5. Ablation Study

We conducted an ablation study for a practical study of the network design. We compared and confirmed our test results to verify the contribution of the mixing module. As mentioned previously, the mixing module helps suppress noise and enhance accurate features, which can be confirmed quantitatively in Table 4. For an unbiased comparison, we trained the network by adding subsequent blocks with the same training parameters. In the beginning, we trained with just the encoder backbone and then we added the feature enhancing block, FEB1, followed by FEB2 and FEB3. After this, we trained the network with the addition of a parallel partial decoder, PPD, and then the mixing module.

In Table 2, we can observe performance improvements with the addition of each block. The baseline network, i.e., the backbone network, already has good performance metrics on all datasets. It has a dice score of 0.899, 0.923, 0.776, 0.878, and 0.753 on Kvasir-SEG, CVC-ClinicDB, CVC-ColonDB, CVC-300, and ETIS, respectively, which is already competitive with the previous best methods. In the next stage, we trained the network with the addition of the FEB1 block and saw a drop in performance in all metrics. After this, we trained the network with the addition of FEB2 to the baseline network. There was a significant improvement in all metrics across all datasets. Similarly, we saw an improvement across all metrics when training the network with the FEB3 block. With the parallel partial decoder, all the features from FEB1, FEB2 and FEB3 were aggregated, and there was a 4% improvement concerning the ETIS dataset over the baseline architecture. The addition of the mixing module exhibits tremendous improvements over the previous module’s output. There are 1.8%, 1.4%, 3.6%, 2.3%, and 5.3% dice score improvements in Kvasir-SEG, CVC-ClinicDB, CVC-ColonDB, CVC-300 and ETIS datasets, respectively, over the baseline architecture. The dice score and mIOU score improvements for the unseen datasets further validate our mixing module network.

**Figure 6:**
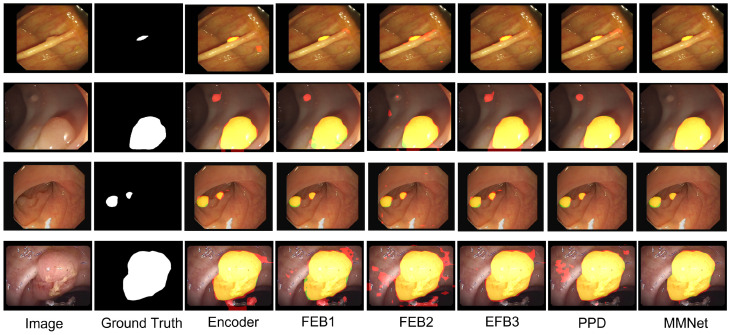
Qualitative analysis of networks with different modules. The green mask is the ground truth, the red mask is a wrongly predicted region and the yellow mask is the GT and predicted overlap region.

This result is also verified qualitatively in Figure 6. Different types of polyps have been used for a comparison. The first is a small polyp where we can observe misidentification by the baseline, FEB1, FEB2, FEB3 and PPD modules. Correct polyp segmentation was achieved upon the addition of the mixing module to the network. The baseline and feature-enhancing blocks with PPD contain various low-level features, so the misidentification and lower dice and mIOU scores are apparent. The addition of a mixing module suppresses such misidentifications. This can be further verified with polyp segmentation across rows two to four. Without the mixing module network, the output either contains unnecessary polyp segmentation or misses the polyp mask. From this, we can observe two roles of the mixing module: the first is removing unnecessary pixels in the segmented mask and the other is restoring the lost pixels that were not segmented in the base model.

## 5. Discussions and Limitations

We studied a medical image segmentation task with various polyp datasets. The main issue with polyp segmentation tasks is the heterogeneity of polyps. The polyps vary in shape, size, color and intensity. Thus, the generalization of various polyp datasets is an arduous task. In this work, five different types of dataset were used; two were used for training purposes, whereas the other three were used for testing the generalization ability. On of the testing datasets contained smaller polyps, which made it even more challenging. A mixing module was introduced as a feature mixer to reintroduce the lost information or discard unnecessary information, and extensive experiments were carried out to confirm its validity. The mixing module was built with depth-wise and 1 × 1 convolution. As seen in Figure 4, the mixing module improved the segmentation accuracy. The results of the proposed approach show improved performance over the traditional methods, which extensively use attention blocks. The proposed approach, especially on the test data, which have been used to test generalization, comfortably surpass the best methods in various evaluation metrics, as seen in Table 1. Furthermore, in this work, an extensive ablation study was carried out to verify the network design, and significant performance improvements can be seen with the addition of mixing blocks.

As shown in Figure 4, the network has difficulty segmenting the image since the polyp and non-polyp regions are very similar. Although our MMNet outperforms the previous best methods, it is still challenging to segment finer, smaller-sized polyps accurately. While our MMNet’s output demonstrates significant improvement over the previous best methods, the segmented mask still loses a few details of large-sized polyps.This implies that while our MMNet outperforms the previous best methods, there is still a need for improvements to restore lost details.

## 6. Conclusions

This paper presents MMNet, a novel multi-stage polyp segmentation network that combines a depth-wise and 1 × 1 convolution model with a pretrained Pyramid Vision Transformer (PVT). By combining these components and incorporating a mixing module to capture global contextual information, our proposed approach achieves remarkable results in polyp segmentation. We have conducted an extensive analysis to verify the effectiveness of our model against various state-of-the-art approaches both qualitatively and quantitatively. Ablation studies have further validated the effectiveness of the mixing module in enhancing the model’s performance. Notably, our proposed approach exhibits a better generalization ability on previously unseen datasets, surpassing the performance of the previous best methods. Specifically, on the challenging ETIS dataset, MMNet achieved a dice coefficient of 0.807, outperforming the previous best method that attained a score of 0.787. These results showcase the potential of our approach to advance the field of polyp segmentation and encourage further research in this direction.

## Figures and Tables

**Table 1:** Quantitative analysis of the proposed approach. The datasets mentioned here were used during training. The upward arrow represents better results with a higher value, whereas the down arrow represents better results with a lower value. Best results are highlighted in bold characters.

Dataset	Methods	mDice ↑	mIOU ↑	$F_{\beta }^{\omega }$ ↑	$S_{\alpha }$ ↑	$E_{\phi }^{\max}$ ↑	MAE ↓
Kvasir-SEG	UNet++ [9]	0.824	0.753	0.808	0.862	0.907	0.048
	SFA [25]	0.725	0.619	0.670	0.782	0.828	0.075
	PraNet [26]	0.901	0.848	0.885	0.915	0.943	0.030
	EU-Net [45]	0.908	0.854	0.893	0.917	0.954	0.028
	MSNet [27]	0.907	0.862	0.893	0.922	0.944	0.028
	UACANet-S [28]	0.905	0.852	0.897	0.914	0.951	0.026
	UACANet-L [28]	0.912	0.859	0.902	0.917	0.958	0.025
	Polyp-PVT [20]	0.917	0.864	**0.911**	0.925	0.962	0.023
	**MMNet (Ours)**	**0.917**	**0.866**	0.910	**0.927**	**0.966**	**0.023**
CVC-ClinicDB	UNet++ [9]	0.797	0.741	0.785	0.872	0.898	0.022
	SFA [25]	0.698	0.615	0.647	0.793	0.816	0.042
	PraNet [26]	0.902	0.858	0.896	0.935	0.958	0.009
	EU-Net [45]	0.902	0.846	0.891	0.936	0.965	0.011
	MSNet [27]	0.921	0.879	0.914	0.941	0.972	0.008
	UACANet-S [28]	0.916	0.870	0.917	0.940	0.968	0.008
	UACANet-L [28]	0.926	0.880	0.928	0.943	0.976	0.006
	Polyp-PVT [20]	0.937	0.889	**0.936**	0.949	0.989	0.006
	**MMNet (Ours)**	**0.937**	**0.889**	0.935	**0.953**	**0.990**	**0.006**

**Table 2:** Quantitative analysis of the proposed approach. The datasets used here were unseen and used only in testing. The upward arrow represents better results with a higher value, whereas the down arrow represents better results with a lower value. Best results are highlighted in bold characters.

Dataset	Methods	mDice ↑	mIOU ↑	$F_{\beta }^{\omega }$ ↑	$S_{\alpha }$ ↑	$E_{\phi }^{\max}$ ↑	MAE ↓
CVC-ColonDB	UNet++ [9]	0.490	0.413	0.467	0.691	0.762	0.064
	SFA [25]	0.467	0.351	0.379	0.634	0.648	0.094
	PraNet [26]	0.716	0.645	0.699	0.820	0.847	0.043
	EU-Net [45]	0.756	0.681	0.730	0.831	0.872	0.045
	MSNet [27]	0.755	0.678	0.737	0.836	0.883	0.041
	UACANet-S [28]	0.783	0.704	0.772	0.848	0.897	0.034
	UACANet-L [28]	0.751	0.678	0.746	0.835	0.878	0.039
	Polyp-PVT [20]	0.808	0.727	0.795	0.865	0.919	0.031
	**MMNet (Ours)**	**0.812**	**0.728**	**0.795**	**0.870**	**0.923**	**0.026**
CVC-300	UNet++ [9]	0.714	0.636	0.687	0.838	0.884	0.018
	SFA [25]	0.465	0.332	0.341	0.640	0.604	0.065
	PraNet [26]	0.873	0.804	0.843	0.924	0.938	0.010
	EU-Net [45]	0.837	0.765	0.805	0.904	0.933	0.015
	MSNet [27]	0.869	0.807	0.849	0.925	0.943	0.010
	UACANet-S [28]	0.902	0.837	0.886	0.934	0.976	0.006
	UACANet-L [28]	**0.910**	**0.849**	**0.901**	0.937	0.980	**0.005**
	Polyp-PVT [20]	0.900	0.833	0.884	0.935	**0.981**	0.007
	**MMNet (Ours)**	0.901	0.834	0.885	**0.938**	0.977	0.006
ETIS	UNet++ [9]	0.413	0.342	0.390	0.681	0.704	0.035
	SFA [25]	0.297	0.219	0.231	0.557	0.515	0.109
	PraNet [26]	0.630	0.576	0.600	0.791	0.792	0.031
	EU-Net [45]	0.687	0.609	0.636	0.793	0.841	0.068
	MSNet [27]	0.719	0.664	0.678	0.840	0.830	0.020
	UACANet-S [28]	0.694	0.615	0.650	0.815	0.851	0.023
	UACANet-L [28]	0.766	0.689	0.740	0.859	0.905	0.012
	Polyp-PVT [20]	0.787	0.706	0.750	0.871	0.910	0.013
	**MMNet (Ours)**	**0.807**	**0.752**	**0.771**	**0.880**	**0.923**	**0.012**

**Table 3:** Comparison of the standard deviation (SD) of the mean dice score (mDice) with different networks. Best results are highlighted in bold characters.

Datasets	Kvasir-SEG	CVC-ClinicDB	CVC-ColonDB	CVC-300	ETIS
**Metrics**	**mDice** ± **SD**	**mDice** ± **SD**	**mDice** ± **SD**	**mDice** ± **SD**	**mDice** ± **SD**
UNet++ [9]	0.821 ± 0.040	0.794 ± 0.044	0.456 ± 0.037	0.707 ± 0.053	0.401 ± 0.057
SFA [25]	0.723 ± 0.052	0.701 ± 0.054	0.444 ± 0.037	0.468 ± 0.050	0.297 ± 0.025
PraNet [26]	0.898 ± 0.041	0.899 ± 0.048	0.712 ± 0.038	0.871 ± 0.051	0.628 ± 0.036
EU-Net [45]	0.908 ± 0.042	0.902 ± 0.048	0.756 ± 0.040	0.837 ± 0.049	0.687 ± 0.039
UACANet-L [28]	0.912 ± N/A	0.926 ± N/A	0.751 ± N/A	**0.910** ± N/A	0.766 ± N/A
Polyp-PVT [20]	0.917 ± 0.042	0.937 ± 0.050	0.808 ± 0.043	0.900 ± 0.052	0.787 ± 0.044
**MMNet (Ours)**	**0.917** ± 0.041	**0.937** ± 0.048	**0.812** ± 0.042	0.901 ± 0.057	**0.807** ± 0.032

**Table 4:** Quantitative analysis of the network’s different modules. An upward arrow represents results where a higher value is better, whereas a down arrow represents results where a lower value is better. Best results are highlighted in bold characters.

Dataset	Methods	mDice ↑	mIOU ↑	$F_{\beta }^{\omega }$ ↑	$S_{\alpha }$ ↑	$E_{\phi }^{\max}$ ↑	MAE ↓
Kvasir-SEG	Backbone	0.899	0.837	0.887	0.912	0.945	0.029
	Backbone + FEB1	0.860	0.783	0.837	0.880	0.923	0.042
	Backbone + FEB2	0.901	0.838	0.884	0.914	0.955	0.031
	Backbone + FEB3	0.906	0.850	0.895	0.918	0.955	0.028
	Backbone + FEB123 + PPD	0.909	0.849	0.896	0.920	0.957	0.026
	**MMNet (Final)**	**0.917**	**0.866**	**0.910**	**0.927**	**0.966**	**0.023**
CVC-ClinicDB	Backbone	0.923	0.868	0.920	0.947	0.989	0.007
	Backbone + FEB1	0.890	0.829	0.880	0.922	0.956	0.017
	Backbone + FEB2	0.905	0.847	0.900	0.930	0.969	0.017
	Backbone + FEB3	0.906	0.846	0.901	0.937	0.973	0.012
	Backbone + FEB123 + PPD	0.919	0.867	0.917	0.942	0.974	0.010
	**MMNet (Final)**	**0.937**	**0.888**	**0.935**	**0.953**	**0.990**	**0.006**
CVC-ColonDB	Backbone	0.776	0.685	0.756	0.850	0.903	0.036
	Backbone + FEB1	0.695	0.603	0.666	0.800	0.859	0.047
	Backbone + FEB2	0.752	0.667	0.730	0.835	0.883	0.044
	Backbone + FEB3	0.783	0.695	0.759	0.853	0.902	0.038
	Backbone + FEB123 + PPD	0.783	0.698	0.764	0.850	0.903	0.037
	**MMNet (Final)**	**0.812**	**0.728**	**0.795**	**0.870**	**0.923**	**0.026**
CVC-300	Backbone	0.878	0.807	0.857	0.928	0.971	0.007
	Backbone + FEB1	0.831	0.738	0.784	0.894	0.965	0.014
	Backbone + FEB2	0.878	0.809	0.855	0.927	0.971	0.008
	Backbone + FEB3	0.869	0.792	0.841	0.919	0.969	0.011
	Backbone + FEB123 + PPD	0.878	0.807	0.853	0.925	0.967	0.011
	**MMNet (Final)**	**0.901**	**0.834**	**0.885**	**0.938**	**0.977**	**0.006**
ETIS	Backbone	0.753	0.663	0.707	0.856	0.908	0.016
	Backbone + FEB1	0.703	0.606	0.652	0.826	0.886	0.020
	Backbone + FEB2	0.748	0.663	0.708	0.858	0.895	0.022
	Backbone + FEB3	0.762	0.674	0.716	0.861	0.894	0.022
	Backbone + FEB123 + PPD	0.790	0.708	0.744	0.878	0.895	0.022
	**MMNet (Final)**	**0.807**	**0.752**	**0.771**	**0.880**	**0.923**	**0.012**

## Data Availability

The training and testing data used in this paper are available at the following links: https://datasets.simula.no/kvasir-seg/ (accessed on 14 November 2022), https://polyp.grand-challenge.org/Databases/ (accessed on 14 November 2022).
